# Research on Engineering the *Saccharomyces uvarum* for Constructing a High Efficiency to Degrade Malic Acid and Low Yield of Diacetyl Biosynthesis Pathway

**DOI:** 10.3390/foods13193161

**Published:** 2024-10-04

**Authors:** Ping Li, Wenjun Song, Shankai Wu, Yumeng Wang, Yicong Fan, Cuiying Zhang

**Affiliations:** 1 College of Biotechnology and Food Science, Tianjin University of Commerce, Tianjin 300134, China; 2 Key Laboratory of Industrial Fermentation Microbiology, Ministry of Education, Tianjin Key Laboratory of Industrial Microbiology, College of Biotechnology, Tianjin University of Science and Technology, Tianjin 300457, China

**Keywords:** wine, diactyl, L-malic acid, flavor balance, heterologous expression

## Abstract

Diacetyl is a flavor compound in wine with a low threshold (1–2 mg/L). It is produced during alcoholic fermentation (AF) *Saccharomyces* and malolactic fermentation (MLF) initiated by lactic acid bacteria (LAB). Whereas, the environment after AF suppresses the normal metabolism of LAB after AF. Researchs have shown the influence on diacetyl mechanisms of the genes *ILV2*, *ILV6*, *ILV3*, *ILV5*, *BDH1*, *BDH2*, and gene *aldB* from *Lactobacillus plantarum* in *Saccharomyce uvarum* WY1. While we found that the diacetyl contents produced by mutants after MLF (Co-fermentation and Seq-fermentation) were significantly improved compared to AF alone. Moreover, the genes *mae1* and *mae2* from *S. pombe*, and gene *mleS* from *L. lactis* exhibited significant effect on deacidification in our previous study, but the diacetyl of the mutants showed obvious improvement in this study. Thus the effects of association mutation of genes (*ILV2*, *ILV6*, *ILV3*, *ILV5*, *BDH1*, *BDH2*, *aldB*, *mae1*, and *mleS*) on deacidification, diacetyl and other flavors (organic acids, higher alcohols and esters) metabolism in *S. uvarum* after AF were detected in the study. Among all the mutants, strains V6AmS, V635mS, and V6B12mS showed the most favorable results. Specifically, the L-malic acid contents decreased to 1.26 g/L, 1.18 g/L, and 1.19 g/L, respectively. Concurrently, diacetyl levels were reduced by 52.56%, 61.84%, and 65.31%. The production of n-propanol increased by 18.84%, 20.89%, and 28.12%, whereas isobutanol levels decreased by 37.01%, 42.36%, and 44.04%, and isoamyl alcohol levels decreased by 19.28%, 19.79%, and 16.74%, compared to the parental strain WY1. Additionally, the concentration of lactate ester in the wine increased to 13.162 mg/L, 14.729 mg/L, and 14.236 mg/L, respectively.

## 1. Introduction

Diacetyl is a crucial flavor component in wine that, at appropriate levels (1–2 mg/L), imparts a pleasant buttery or cheesy flavor, contributing to the wine’s texture and aroma. However, concentration at higher level can produce undesirable “buttery” off-flavor. During fermentation, diacetyl is produced by yeast cells in the early stages AF and by LAB in the later stages MLF, resulting in levels of diacetyl that can easily exceed threshold value in wine [1,2,3,4,5,6]. Therefore, adequate control of diacetyl levels is crucial for improving the flavor quality of wine.

**Figure 1:**
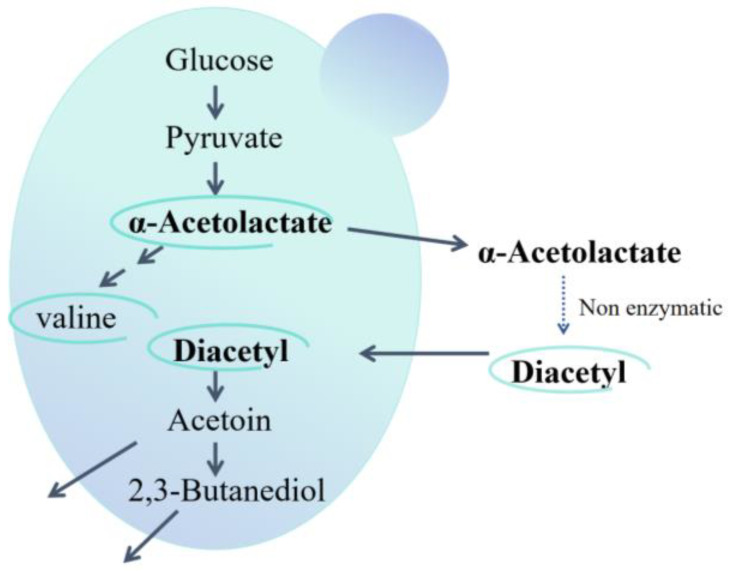
The diacetyl metabolic pathway in yeast.

**Figure 2:**
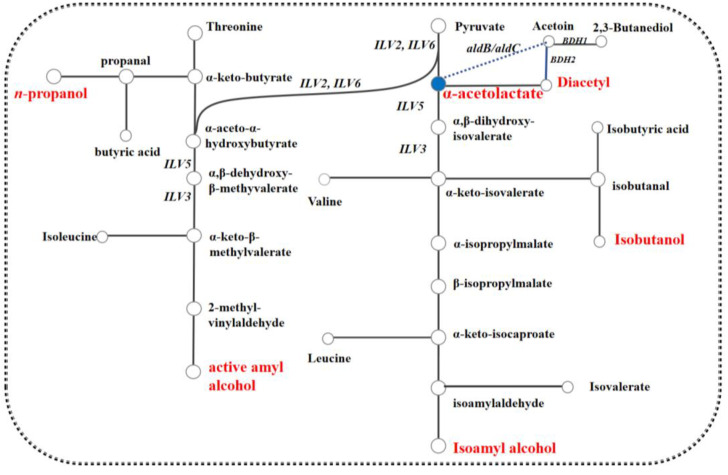
The metabolic pathway of higher alcohols (the dotted line represents the pathway created by the introduction of the exogenous gene *aldB*/*aldC* encoding α-acetolactate decarboxylase).

During AF, α-acetolactate is synthesized from pyruvate by α-acetolactate synthase and subsequently secreted into the extracellular space, where it is degraded to diacetyl through non-enzymatic oxidation and decarboxylation. The diacetyl is then reabsorbed into the cell and reduced to acetoin and 2,3-butanediol by diacetyl reductase and 2,3-butanediol dehydrogenase as shown in Figure 1. Meanwhile, some pyruvate and α-acetolactate can be converted into isoleucine, leucine, and valine through the actions of hydroxyacid reductase and 2,3-dihydroxyisovalerate dehydratase in the isoleucine-leucine-valine (ILV) metabolic pathway [7,8,9]. Researches on diacetyl regulation in AF have predominantly focused on the brewing industry, with less attention given to wine fermentation. For example, Shi Tingting et al. knocked out one *ILV2* (encoding α-acetolactate synthase) allele and overexpressed genes *BDH1* (encoding 2,3-butanediol dehydrogenase) and *BDH2* (encoding diacetyl reductase), achieving a 58.70% decrease in diacetyl content [10,11]. Choi. and Cejnar. heterologously expressed the α-acetolactate decarboxylase gene *aldC* from *Enterobacter aerogenes* and *Acetobacter aceti* respectively, resulting in notably lower diacetyl production [12,13]. As *S. uvarum* could exhibit notable oenological properties, including higher glycerol production and lower ethanol levels, as well as greater aromatic intensity compared to those of *S. cerevisiae* [14,15,16]. In previous research, we investigated the effect of genes *ILV2*, *ILV6*, *ILV3*, *ILV5*, *BDH1*, *BDH2*, *aldB* from *L. plantarum* in *S. uvarum* on diacetyl mechanism during wine fermentation. We found that the diacetyl content produced by the mutants significantly decreased compared to the parental strain after AF in wine [17,18,19]. However, these studies mainly target diacetyl alone and overlook the effects on other metabolites, such as higher alcohols, as well as the overall flavor balance (Figure 2). And following further MLF, the diacetyl levels were still prone to exceed the threshold range for diacetyl in wine.

MLF is a process in which LAB convert sharp, malic acid compounds into softer, stable lactic acid in wine. Whereas, environment with the higher alcoholic strength, lower pH, and less residual sugar levels from the completion of AF inhibit the normal metabolic processes of LAB, thereby delaying MLF, prolonging the fermentation period, and leading to the uncontrollable growth of bacteria that generate off-flavors and odors [20,21,22,23,24]. Researches had focused on cloning genes associated with malic acid reduction in LAB or other strains and expressing them in *S. cerevisiae*, leading to the development of the *S. cerevisiae* capable of both AF and significant deacidification reduction, and simplifying the fermentation process [25,26,27,28,29]. For example, Husnik et al.were able to construct a stable genetically engineered wine *S. cerevisiae*, carrying the *Sz pombe* mae1 and the *Oenococcus oeni* malolactic gene *mleA*, resulting in fully decarboxylated 5.5 g/l of malate [30]. constructed recombinant *S. cerevisiae* with expression of malate transporter (Mae1) and fumarase genes improved the malic acid conversion significantly [31]. In previous study, different genes encoding malic enzyme, malolactic enzyme, and malate permease from *several LAB* and *Schizosaccharomyces pombe*, were heterologously expressed and displayed differential impact on malic acid and higher alcohols metabolism in *S. uvarum*. Among these, strain WYm1S with co-expression of the malate permease gene *mae1* from *S. pombe* and the malolactic enzyme gene *mleS* from *L. lactis*, and WYm1m2 with co-expression of gene *mae1* and malate permease gene *mae2* from *S. pombe* were selected and bred for their ability to reduce L-malic acid content to approximately 1 g/L [32]. Whereas the fermentation of WYm1m2 resulted in an increase in the levels of higher alcohols (such as isoamyl alcohol, n-propanol), which, when present in excess, can affect the taste of the wine and pose risks to human health. And the effect of the genes on diacetyl metabolism in *S. uvarum* remained to be seen. Moreover, regulating one metabolite during fermentation inevitably affects the stability of other flavor compounds. Therefore, achieving an optimal level of diacetyl while maintaining a harmonious flavor profile is essential for enhancing the quality and character of the wine.

In this study, we constructed biological pathway of deacidification and low-yield diacetyl into *S. uvarum* through association mutation of genes *ILV2*, *ILV6*, *ILV3*, *ILV5*, *BDH1*, *BDH2*, *aldB* from *L. plantarum*, *mae1* and *mae2* from *S. pombe*, and *mleS* from *L. lactis* to investigate the effect of the genes association mutation on the diacety, malic acid, and other flavor (organic acids, higher alcohols and esters) metabolism. Additionally, physiological characteristics and fermentation performance, including growth curves, fermentation rates, ethanol content, residual sugar production in wine, were analyzed to confirm the synergistic effects of the genes in *S. uvarum*.

## 2. Material and Methods

### 2.1. Strains and Media

The strains used in this study, including *Escherichia coli* DH5α, *S. uvarum*, *L. lactis*, and *S. pombe* were detailed in Table 1.

The *E. coli* DH5α strain, used as a host for recombinant DNA manipulation, was grown in LB broth (1% NaCl, 1% tryptone, 0.5% yeast extract) at 37 °C and 200 rpm for about 12 h. Appropriate antibiotics were added for plasmid construction at final concentrations of 100 mg/L ampicillin or 100 mg/L kanamycin. All of the solid media used in this study contained 2% agar

*L. lactis* was cultured in SGM17 medium (37.25 g/L M17 broth, 5 g/L sucrose, and 5.5 g/L glucose) at 30 °C for about 10 h. *L. plantarum* was propagated in De Man–Rogosa–Sharpe (MRS) medium (2% glucose, 1.0% tryptone, 1.0% beef extract, 1.0% yeast extract, 0.2% K_2_HPO_4_, 0.1% Tween-80, 0.02% MgSO_4_·7H_2_O, 0.005% MnSO_4_·4H_2_O, 0.2% ammonium citrate, 0.5% sodium acetate, 50 mM NaHCO_3_, pH 5.0 adjusted with HCl) at 37 °C for about 10 h. All of the solid media used in this study contained 2% agar

*S. uvarum* and *S. pombe* were grown in YPD medium (20 g/L glucose, 10 g/L yeast extract, 20 g/L bacto-peptone) at 28 °C and 180 rpm for 12 h. All engineered strains were derivatives of *S. uvarum* WY1. Yeast cultures were supplemented with 200 mg/L G418 to select for Geneticin-resistant strains following transformation. For Cre expression in transformants, *S. uvarum* was grown in yeast extract peptone glucose (YEPG) medium (1% yeast extract, 2% peptone, 2% galactose). To select for Zeocin-resistant strains, 500 mg/L Zeocin (Promega, Madison, WI, USA) was added to YEPD plates during culture. All solid media in this study contained 2% agar.

### 2.2. Construction of Plasmids and Mutant Strains

The plasmids used in this study, including Yep352, pUC19-PGK, and pUG6, are listed in Table 1. Plasmids pUC19-PGK and pUG6 served as templates to obtain the promoter *PGK1* fragment and the selection marker *KanMX* fragment, respectively.

The target fragments GAL80A, GAL80B, V2m1SA, and V2m1SB were amplified using PCR with primers GAL80A-U/GAL80A-D, GAL80B-U/GAL80B-D, V2m1SA-U/V2m1SA-D, and V2m1SB-U/V2m1SB-D, using *S. uvarum* WY1 as the template. Additionally, we used the primers ReGAL80-U/ReGAL80-D and ReV2m1S-U/ReV2m1S-D, with the plasmid Yep-Pm1SNZK as the template, to amplify long fragments containing PGKp-mae1-PGK, PGKp-mleSNZ-PGK, and KanMX. These fragments were named ReGAL80 and ReV2m1S, respectively. Subsequently, the fragments GAL80A, GAL80B, and ReGAL80 were simultaneously introduced into the *V26* strain using lithium acetate/PEG method as previous, resulting in the mutant strain V6mSK [17]. Similarly, the fragments V2m1SA, V2m1SB, and ReV2m1S were simultaneously introduced into the V22 strain, resulting in the mutant strain V2mSK. The Cre-loxP recombination system, combined with a gene disruption strategy, was used to rescue the *KanMX* gene in V2mSK and V6mSK, resulting in the mutants V2mS and V6mS, respectively [33]. The plasmids Yep-PAK, Yep-PV35K, and Yep-PB12K were introduced into the mutant *V2mS*, leading to the recombinant strains V2AmS, V235mS, and V2B12mS. Similarly, these plasmids were introduced into the mutant *V6mS*, resulting in the recombinant strains V6AmS, V635mS, and V6B12mS. (Table 1 and Table 2).

### 2.3. Fermentation Conditions

Early-stage preparations: Fresh grape grapes were harvested, destemmed, and crushed at optimal technological maturity. Subsequently, 80 mg/L SO_2_ was added to inhibit indigenous microflora. The must was stored overnight at 4 °C, and then was adjusted to 20.45° Brix by adding sucrose, and the pH was adjusted to 3.4–3.6 using tartaric acid. The *S. uvarum* strains were maintained in YEPD medium at 28 °C and 180 rpm. After a 12-h incubation, the yeast cells were subcultured into 50 mL of YEPD medium under similar conditions. Similarly, *L. plantarum* strains were maintained in MRS medium at 37 °C. After a 10-h incubation, the cells were subcultured into 50 mL of YEPD medium under similar conditions. Bacterial sludge was harvested by centrifugation (5000 rpm, 4 °C, 5 min) and washed once with 50 mL of sterile water.

AF: 250 mL fifty flasks, autoclaved at 121 °C for 15 min before use, were filled with 180 mL of the formulated grape juice. The *S. uvarum* cells was diluted to the counts of 1 × 10^8^ CFU/mL, and added to the flasks to activate AF. The grape must after inoculation was maintained at 25 °C in a temperature-controlled room and weighed every 12 h to monitor fermentation progress until the daily weight loss was less than 0.3 g, indicating that fermentation was complete. All fermentations were conducted in triplicate.

Seq-fermentation: After AF, the mixture was skimmed to remove scum under aseptic conditions and then transferred to similar bottles. *L. plantarum* cells were diluted to 1 × 10^7^ CFU/mL, added to the bottles, and fermented at 18 °C to initiate MLF. Malic acid content was measured every 2 days until it stabilized, indicating that fermentation was complete. All fermentations were conducted in triplicate.

Co-fermentation: At the beginning of AF, the microorganisms were prepared with cell counts of 1 × 10^8^ CFU/mL for *S. uvarum* and 1 × 10^7^ CFU/mL for *L. plantarum*, and added to the flasks simultaneously. The grape must was fermented at 25 °C until the malic acid content stabilized, indicating the completion of fermentation. All fermentations were conducted in triplicate.

### 2.4. Chemical Analysis

Diacetyl in wine was detected using high-performance liquid chromatography with ultraviolet detection (HPLC-UV) coupled with pre-column derivatization using the reagent 3,3′-diaminobenzidine (DAB). A Shim-pack VP-ODS column (250 mm × 4.6 mm, 4.6 µm) was employed with water-methanol mobile phases for gradient elution at a flow rate of 0.7 mL/min and a detection wavelength of 254 nm.

Malic acid, L-lactic acid, and other organic acids were identified by high-performance liquid chromatography (HPLC) which was performed using an Aminex HPX-87H column with 5 mmol/L H_2_SO_4_ as the mobile phase, at a flow rate of 0.6 mL/min and a temperature of 65 °C.

Flavor compounds, including higher alcohols, ethyl acetate, and ethyl lactate, were analyzed using an Agilent 7890C gas chromatograph (GC) in which butyl acetate was used as the internal standard, with nitrogen (purity ≥ 99.99%) serving as the carrier gas at a constant flow rate of 0.8 mL/min. The injector temperature was set to 200 °C, and the oven temperature program began at an initial temperature of 50 °C for 8 min, followed by an increase to 150 °C at a rate of 5 °C/min for 15 min.

The growth curves of the parental and mutant strains were monitored using the Bioscreen Automated Growth Curves Analysis System. CO_2_ weight loss was measured at 12-h intervals using an analytical balance throughout the fermentation process. Following fermentation, pH values, ethanol concentrations, and residual sugar contents were determined with a pH meter, an oenometer, and a Brix hydrometer, respectively. All analyses were performed in triplicate.

### 2.5. Statistical Analysis

Data were represented as the mean ± standard errors. The differences between the mutant strains and parental strain were confirmed by Student’s *t*-test. Statistical significance was considered at *p* < 0.05.

## 3. Results and Discussion

### 3.1. The Effect of MLF on Diacetyl Production during Mutant S. uvarum with Low Yield of Diacetyl Fermentation

**Figure 3:**
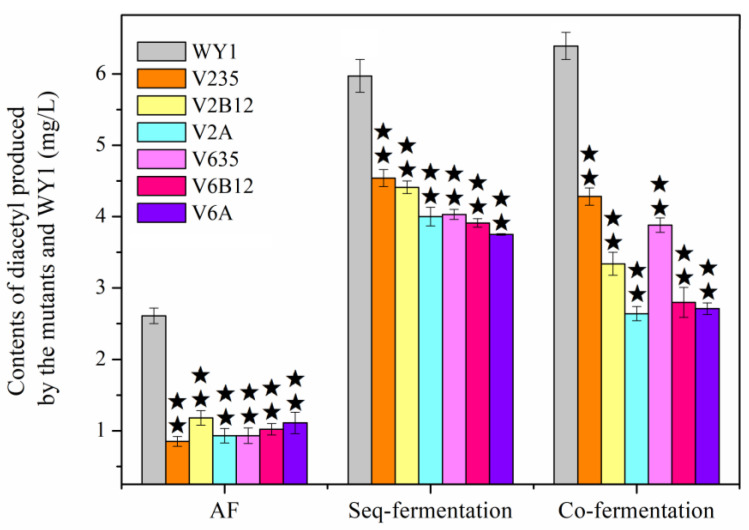
The diacetyl concentration produced by the mutants after AF, Seq-fementation, and Co-fementation respectively (Error bars represent the SD of the average values. Statistical significance is denoted as ★★ = *p* < 0.01).

Researches have shown that the *ILV2*, *ILV6*, *ILV3*, *ILV5*, *BDH1*, *BDH2*, and *L. plantarum* aldB genes affect diacetyl metabolism in *S. uvarum* [17,18,19], leading to the construction of corresponding low-diacetyl *S. uvarums* V235 (with *ILV2* deletion and *ILV3* + *ILV5* over-expression), V2B12 (with *ILV2* deletion and *BDH1* + *BDH2* over-expression), V2A (with *ILV2* deletion and *aldB* from *L. plantarum* heterologous expression), V635 (with *ILV6* deletion and *ILV3* + *ILV5* over-expression), V6B12 (with *ILV6* deletion and *BDH1* + *BDH2* over-expression), V6A (with *ILV6* deletion and *aldB* from *L. plantarum* heterologous expression). To further investigate the effect of MLF on diacetyl production, the diacetyl contents detected after seq-fermentation (AF + MLF) and co-fermentation in mixed cultures of *S. uvarum* and *L. plantarum* (AF + MLF) compared with by AF alone. As Figure 3 shown. The diacetyl contents produced by mutant strains V235, V2B12, V2A, V635, V6B12 and V6A were decreased 67.56%, 54.63%, 64.34%, 64.18%, 60.74%, 57.62%, respectively, compared with the parental strain WY1 (2.61 mg/L, *p* < 0.05) after AF (about 5 days). And the diacetyl contents produced by mutant strains during seq-fermentation (about 25 days) were decreased by 23.86%, 26.00%, 32.94%, 30.21%, 32.14% and 35.01%, respectively, compared with the parental strain WY1 (5.97 mg/L, *p* < 0.05); And the diacetyl production by the mutant strains after co-fementation (about 15 days) were decreased by 34.55%, 47.79%, 58.76%, 39.24%, 56.22% and 57.66%, respectively, compared with the parental strain (6.39 mg/L, *p* < 0.05).

It was demonstrated that, combining the inhibition of precursor production with the acceleration of α-acetolactate metabolism into other compounds or the enhancement of diacetyl reduction pathways was more effective than single-pathway mutations in diacetyl reduction during AF, Seq-fermentation, or Co-fermentation. During yeast alcoholic fermentation, Ilv2p and Ilv6p catalyze the formation of α-acetyl-lactate from pyruvate. Some α-acetyl-lactate undergoes non-enzymatic oxidative decarboxylation to form diacetyl. Diacetyl is then converted into ethyl diphthongs and 2,3-butanediol by diacetyl reductase Bdh2p and 2,3-butanediol dehydrogenase Bdh1p. Additionally, some α-acetyl-lactate is oxidatively decarboxylated by hydroxylate reductase Ilv5p, with the aid of enzymes such as dihydroxyisovalerate dehydratase Ilv3p, to produce valine and isoleucine. The knockout of the *ILV6* or *ILV2* genes lead to a reduction in the expression of the corresponding genes and a decrease in acetolactate synthase, thereby hindering the formation of the diacetyl precursor α-acetolactate [10,11]. Meanwhile, under the control of the *PGKp* promoter, the expression levels of *ILV3* + *ILV5* or *BDH1* + *BDH2* were significantly increased [17,18]. The overexpression of *ILV3* + *ILV5* accelerated the conversion of α-acetolactate to leucine and valine, as well as the metabolism of pyruvate to α-ketoisocaproate, effectively reducing the conversion of α-acetolactate to diacetyl. And, the overexpression of *BDH1* + *BDH2* enhanced the metabolic flow from diacetyl to acetoin and 2,3-butanediol, further reducing diacetyl levels. Additionally, the *aldB* gene, encoding α-acetolactate decarboxylase, catalyzed the direct conversion of α-acetolactate to acetoin, thereby further decreasing the metabolic flow from α-acetolactate to diacetyl. It is interesting that, the mutant strains had a slightly greater impact on diacetyl metabolism during co-fermentation compared to seq-fermentation. The residual low levels of nutrients reduce the *S. uvarum*’s fermentation capacity during the later stages of Seq-fermentation, resulting in a slightly smaller impact on diacetyl production. Besides this, This effect was attributed to the effect of the *ILV3*, *ILV5*, and *aldB* genes on α-acetolactic acid metabolism during co-fermentation, the degradation of diacetyl by the overexpression of *BDH1* and *BDH2* genes, maybe, and potentially the interactions between the metabolisms of *S. uvarum* and *L. plantarum* [5,7,10,11,19]. In spite of this, the diacetyl contents after LAB fermentation still showed a significant increase, exceeding the threshold for diacetyl in wine. Therefore, it is necessary to eliminate the impact of LAB fermentation on diacetyl production.

### 3.2. The Effect on Diacetyl of Genes Related L-Malic Acid Matebolism Heterologous Expression in S. uvarum

**Figure 4:**
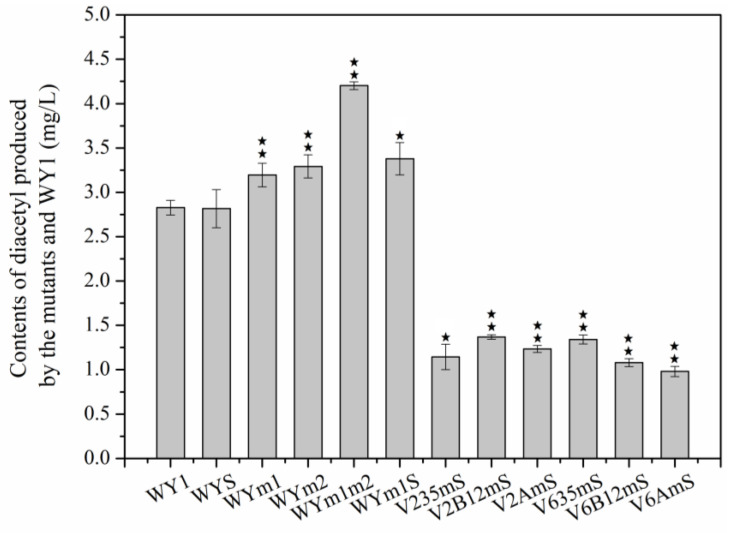
The diacetyl contents produced by WY1 and the mutants after AF (Error bars represent the SD of the average values. Statistical significance is denoted as ★★ = *p* < 0.01, ★ = *p* < 0.05).

**Figure 5:**
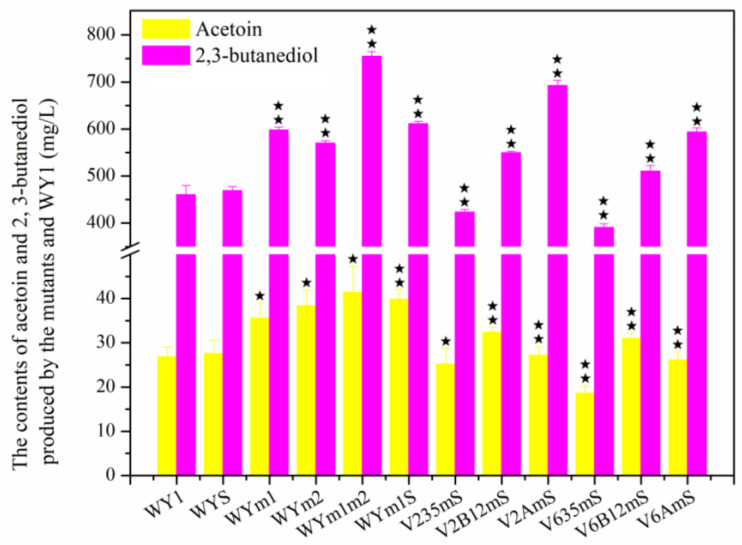
The acetoin and 2,3-butanediol contents produced by WY1 and the mutants after AF (Error bars represent the SD of the average values. Statistical significance is denoted as ★★ = *p* < 0.01, ★ = *p* < 0.05).

The winemaking process generally consists of AF, carried out by yeast, and MLF, in which LAB decarboxylate and degrade the dibasic acid L-malic acid in the wine to the monobasic acid L-lactic acid and CO_2_. However, uncontrolled fermentation by lactic acid bacteria not only adversely affects the normal flavor of the wine but also increases diacetyl levels. In a previous study, we found that the strains WYm1S, which co-expressed gene *mae1* from *S. pombe* and gene *mleS* from *L. lactis*, and WYm1m2, which co-expressed genes *mae1* and *mea2* from *S. pombe*, reduced L-malic acid content to approximately 1 g/L. To investigate the impact of heterologous genes on diacetyl metabolism, the concentrations of diacetyl and downstream products, acetoin and 2,3-butanediol after AF were measured in mutants WYm1 (with heterologous expression of *mae1* from *S. pombe*), WYm2 (with heterologous expression of *mea2* from *S. pombe*), WYS (with heterologous expression of *mleS* from *L. lactis*), WYm1S, and WYm1m2 fermentation. As shown in Figure 4, diacetyl contents in mutants WYm1, WYm2, WYm1m2, and WYm1S were 2.95 mg/L, 3.04 mg/L, 3.88 mg/L, and 3.12 mg/L, respectively, which represent increases of 13.03%, 16.48%, 48.66%, and 19.54% compared to the parental strain WY1 (2.61 mg/L, *p* < 0.05). As diacetyl levels increased, so did the levels of acetoin and 2,3-butanediol as shown in Figure 5 (*p* < 0.05). The result demonstrated that the heterologous expression of genes related L-malic acid matebolism had significant effect on diacetyl metabolism in *S. uvarum*.

The malic enzymes *Mae2* in *S. pombe* are responsible for catalyzing the oxidative decarboxylation of L-malate to pyruvate and CO_2_. Malic acid remained at a high level during the early stage of AF. Thus the gene *mae2* heterologous expression in *S. uvarum* promoted the synthesis of more pyruvate from malic acid [32,34]. And then pyruvate is further metabolized to produce higher levels of diacetyl and other metabolites such as acetoin and 2,3-butanediol. Malate permease Mae1 in *S. pombe* are active transporters of malate from extracellular to intracellular compartments [34]. As the the gene *mae1* heterologous expression in *S. uvarum*, the native malic enzymes *MAE1* in *S. uvarum* and/or malic enzymes *Mae2* from *S. pombe* further metabolizes malic acid to pyruvate, resulting in increased diacetyl content. That is say, heterologous expression of genes in *S. uvarum* could eliminate the destructive impact of LAB fermentation and reduced L-malic acid content, but it also led to increased diacetyl levels in wine. Thus, it is necessary to construct the strain with a ability of high efficiency to degrade malic acid and low yield of diacetyl.

### 3.3. Effects of Association Mutation of Genes in S. uvarum on Diacetyl and L-Malic Acid Yields Production

As the mutant WYm1S had a smaller effect on diacetyl, and better effect on the flavor quality improvement compared to WYm1m2 [32], we cnstructed the biological pathway of deacidification and low-yield diacetyl into *S. uvarum* with association mutation of genes related to diacetyl metabolism (*ILV2*, *ILV6*, *ILV3*, *ILV5*, *BDH1*, *BDH2*, *aldB* from *L. plantarum*) and genes *mae1* from *S. pombe* and *mleS* from *L. lactis*, obtaining the mutants V235mS, V2B12mS, V2AmS, V635mS, V6B12mS, V6AmS. The levels of diacetyl and L-malic acid after AF by the mutants and the parental strain WY1 were measured to investigate the effects of the association mutation of genes on diacetyl and L-malic acid metabolism in *S. uvarum*.

As shown in Figure 4, the diacetyl contents after AF by strains V235mS, V2B12mS, V2AmS, V635mS, V6B12mS, and V6AmS were 1.14 mg/L, 1.37 mg/L, 1.24 mg/L, 1.34 mg/L, 1.08 mg/L, and 0.98 mg/L, respectively. These values represent reductions of 59.53%, 51.61%, 56.31%, 52.56%, 61.84%, and 65.31% compared to strain WY1 (*p* < 0.05). Furthermore, with changes in diacetyl contents, the levels of acetoin and 2,3-butanediol also fluctuated significantly as shown in Figure 5. The acetoin levels produced by strains V2B12mS and V6B12mS were 32.38 mg/L and 30.97 mg/L, respectively, which were 20.65% and 15.42% higher than that of the parental strain WY1 (26.83 mg/L, *p* < 0.05), The 2,3-butanediol production after fermentation of strains V2AmS, V6AmS, V2B12mS, and V6B12mS were 47.97%, 28.88%, 19.32%, and 10.78% higher than that of the starting strain WY1 (460.36 mg/L, *p* < 0.05). Conversely, the acetoin levels in the fermentation broths of strains V235mS and V635mS showed 6.29% and 30.72% lower than that of the parental strain WY1 (*p* < 0.05). And the 2,3-butanediol content in these strains exhibited 8.11% and 15.20% lower than that of parental strain WY1 (*p* < 0.05). Although the heterologous expression of the mae1 gene shifts malate from the extracellular to the intracellular compartment, and the native malic enzyme in *S. uvarum* converts malate to pyruvate, the reduction in acetyl lactate synthase activity and the enhancement of dehydroxyacid dehydratase and ketol-acid reductoisomerase activities could shift the metabolic flow away from diacetyl, resulting in decreased production of acetoin and 2, 3-butanediol in wine by V235mS and V635mS fermented [10,11,12,13]. During V2AmS, V6AmS, V2B12mS, and V6B12mS fermentation, the metabolic flow for diacetyl degradation may be greater than that for α-acetolactate synthesis. Meanwhlie, heterologous expression of the *aldB* gene directly converts α-acetolactate, produced from pyruvate, to acetoin. Additionally, the overexpression of genes *BDH1* + *BDH2* accelerated the catalysis of diacetyl reductase and 2,3-butanediol dehydrogenase, resulting in decreased diacetyl levels and higher production of acetoin and 2,3-butanediol [19,35]. It could also be because of interactions between genes and metabolic streams which will be further discussed by several method such as metabonomics analysis combined with transcriptomics in our later research.

**Figure 6:**
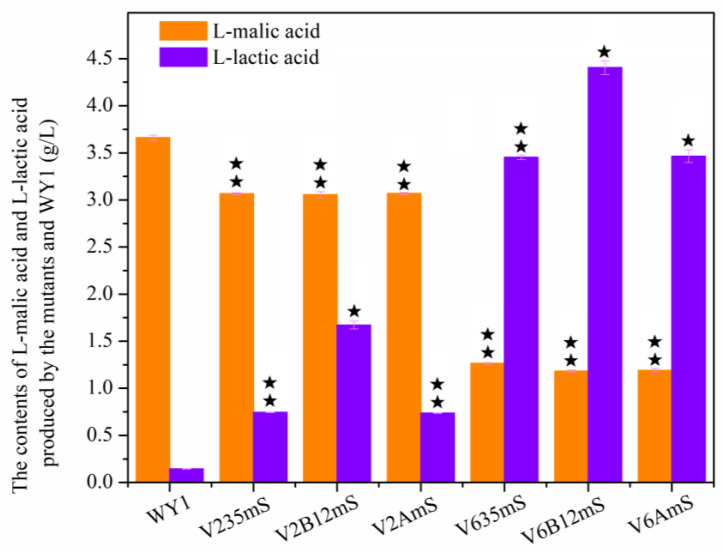
The contents of L-malic acid and L-lactic acid produced by WY1 and the mutants with deacidification and low-yield diacetyl genes after AF (Error bars represent the SD of the average values. Statistical significance is denoted as ★★ = *p* < 0.01, ★ = *p* <0.05).

The Figure 6 showed that the L-malic acid content in the fermentation broth after AF by the mutant strains V235mS, V2B12mS, V2AmS, V635mS, V6B12mS, and V6AmS were 3.07 g/L, 3.06 g/L, 3.07 g/L, 1.27 g/L, 1.18 g/L, and 1.19 g/L, respectively, representing reductions of 16.22%, 16.51%, 16.16%, 65.43%, 67.65%, and 67.49% (*p* < 0.05) compared to the starting strain WY1 (3.661 g/L). In contrast, the L-lactic acid content increased by 4.17-fold, 10.61-fold, 4.12-fold, 22.98-fold, 29.59-fold, and 23.06-fold (*p* < 0.05), respectively. It is demonstrated that, heterologous expression of *mae1*, a malate permease gene from *S. pombe*, and *mleS*, a malate lactamase gene from *L. lactis*, in mutants V2AmS, V235mS, and V2B12mS resulted in a slight reduction in the L-malate content of the corresponding fermentation broths. This may be due to the knockout of gene *ILV2* or gene interactions in *S. uvarum* affecting the normal expression of the exogenous genes *mae1* and *mleS* compared to those in strain WYmS. To test this hypothesis, we measured the relative expression levels of *mae1* and *mleS* in strains V2AmS, V235mS, and V2B12mS, which were significantly reduced compared to strain WYmS. Specifically, the expression levels of *mae1* in V2AmS, V235mS, and V2B12mS decreased by 62%, 59%, and 56%, respectively, compared to WYmS. Similarly, the expression levels of *mleS* decreased by 42%, 45%, and 52%, respectively, compared to WYmS (*p* < 0.05). Whereas, the expression of *mae1* and *mleS* in mutants V6AmS, V635mS, and V6B12mS did not alter the impact of these genes on L-malate degradation compared to WYmS as previous study [32]. The L-malic acid levels in the mutants were all reduced to approximately 1 g/L within 5 days of AF, The result was also in conjunction with the report by Bony et al.who obtained a malolactic *S. cerevisiae* through the co-expression of the *Sz. pombe* malate transport gene (*mae1*) and the *L. lactis* malolactic enzyme gene (*mleS*) under the control of promoter *PGK1*, leading to efficient decarboxylation 3 g/L of malate within a 3-day period [36], which also demonstrated that the mutation of genes *ILV6*, *ILV3*, *ILV5*, *BDH1*, *BDH2*, and *aldB* did not affect the regulation on malic acid metabolism of genes *mae1* and *mleS*. Moreover, it is noteworthy that the L-lactic acid contents in V2B12mS and V6B12mS were significantly higher than that in other related mutants after fermentation. This increase may be due to the overexpression of the *BDH1* and *BDH2* genes affecting L-lactic acid metabolism or the impact of *mleS* on L-malic acid, and further exploration is needed.

### 3.4. Effects of Association Mutation of Genes in S. uvarum on Higher Alcohols and Esters Production

**Figure 7:**
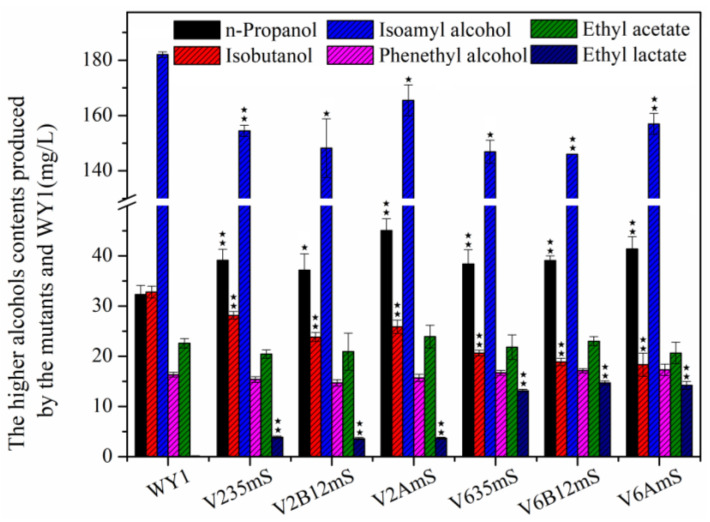
The contents of higher alcohols produced by WY1 and the mutants with deacidification and low-yield diacetyl genes after AF (Error bars represent the SD of the average values. Statistical significance is denoted as ★★ = *p* < 0.01, ★ = *p* < 0.05).

Higher alcohols and esters are crucial flavor compounds in wine. To investigate the effects of the association mutation of genes in *S. uvarums* on flavor in wine, the concentration of higher alcohols and esters were detected after AF by the mutants V235mS, V2B12mS, V2AmS, V635mS, V6B12mS, and V6AmS. As shown in Figure 7, the production of phenethyl alcohol and ethyl acetate by mutant strains V235mS, V2B12mS, V2AmS, V635mS, V6B12mS, and V6AmS was similar to those of the parent strain WY1. However, the contents of n-propanol in the mutants increased, and the levels of isobutanol and isoamyl alcohol decreased significantly. Namely, the n-propanol concentrations in the products from V235mS, V2B12mS, V2AmS, V635mS, V6B12mS, and V6AmS were 39.14 mg/L, 37.16 mg/L, 45.05 mg/L, 38.41 mg/L, 39.07 mg/L, and 41.41 mg/L, respectively. These represent increases of 21.09%, 14.98%, 39.45%, 18.84%, 20.89%, and 28.12% compared to the parent strain WY1 (32.22 mg/L, *p* < 0.05). In contrast, isobutanol concentrations were 28.16 mg/L, 23.86 mg/L, 25.88 mg/L, 20.66 mg/L, 18.90 mg/L, and 18.35 mg/L, reflecting reductions of 14.12%, 27.24%, 21.08%, 37.01%, 42.36%, and 44.04% compared to WY1 (32.79 mg/L, *p* < 0.05). Similarly, isoamyl alcohol levels were 154.46 mg/L, 154.71 mg/L, 165.48 mg/L, 146.90 mg/L, 145.97 mg/L, and 151.52 mg/L, showing reductions of 15.12%, 14.98%, 9.07%, 19.28%, 19.79%, and 16.74% compared to WY1 (181.98 mg/L, *p* < 0.05). Additionally, lactate ester levels in the fermented products from V235mS, V2B12mS, V2AmS, V635mS, V6B12mS, and V6AmS increased to 3.882 mg/L, 3.603 mg/L, 3.70 mg/L, 13.16 mg/L, 14.73 mg/L, and 14.24 mg/L, respectively, while the lactate ester content in WY1 was nearly zero.

Previous studies demonstrated that the gene *ILV2*, *ILV6*, *ILV3*, *ILV5*, and *aldB* from *L. plantarum* play significant roles in the metabolism of higher alcohols during *S. uvarum* fementation. The deletion of *ILV2*/*ILV6* prevented the conversion of pyruvate to α-acetolactate, thereby effectively increasing the metabolic flux from pyruvate to α-ketoisovalerate and Pyruvate, in which α-Ketoisovalerate is converted to n-propanol through decarboxylation and reduction, while Pyruvate is metabolized to ethanol and acetic acid by alcohol dehydrogenase and aldehyde dehydrogenase, respectively. Ethanol and acetic acid then react to form ethyl acetate [10,11,12,13,14]. The inhibition of α-acetolactate synthesis results in decreased levels of isobutanol and isoamyl alcohol, which are typically produced from α-acetolactate by hydroxyacid reductase and dehydratase. Additionally, α-acetolactate can be converted to α-ketoisovalerate by Ilv5p and Ilv3p. α-Ketoisovalerate is then further converted to α-isopropylmalate and subsequently to α-ketoisocaproate through the actions of α-isopropylmalate dehydrogenase and β-isopropylmalate dehydrogenase, respectively. Both α-ketoisovalerate and α-ketoisocaproate are decarboxylated and reduced to isobutanol and isoamyl alcohol, respectively, while Ilv3p and Ilv5p also catalyze the conversion of α-ketoisovalerate to active pentanol through a series of reactions. Consequently, overexpression of *ILV3* and *ILV5* significantly increases isobutanol levels while decreasing the metabolic flux from α-ketoisovalerate to n-propanol, leading to reduced n-propanol content in the fermentation broth of the corresponding mutant strains Furthermore, the *aldB* gene from *L. plantarum* encodes acetolactate decarboxylase, which directly converts α-acetolactate to acetoin, which effectively reduces the metabolic flux from α-acetolactate to α-ketoisovalerate and α-ketoisocaproate, thereby lowering the levels of isobutanol and isoamyl alcohol (as shown in Figure 7) [5,7,18].

The malate transporter facilitates the transfer of L-malate and α-ketoglutarate from the extracellular environment to the intracellular space, promoting the expression of malic enzyme and malolactamase genes, which lead to the degradation of malic acid into lactic acid or pyruvic acid. And the malolactic enzyme encoded by the genes *mleS* or *mleA* degrades malic acid to lactic acid, which then reacts with ethanol to produce ethyl lactate. This process effectively slows the metabolic flow from malic acid to pyruvic acid in *S. uvarum* [28,29,30,32,36]. Thus the synergistic effect and interactions of the genes resulted in different trends in the production of higher alcohols and esters after AF. The n-propanol contents produced by mutant strains V635mS, V6B12mS, and V6AmS were higher than that of the parent strain WY1. In contrast, the n-propanol production from low-yielding diacetyl strains V635, V6B12, and V6A, as well as from the L-malic acid-reducing strain WYm1S, were all comparable to that of the parent strain. This discrepancy may be due to the influence of the exogenous genes mae1 and mleS on the selective permeability of α-ketoglutarate, thereby affecting n-propanol metabolism in strains V635mS, V6B12mS, and V6AmS. The expression of genes *mae1* and *mleS* led to reduction in isobutanol, isoamyl alcohol, and ethyl acetate levels in our previous study. And strains V635, V6B12, and V6A showed decreased isobutanol and isoamyl alcohol production, but increased ethyl acetate levels, indicating that the synergistic effect of mae1 and mleS expression in these strains resulted in reduced isobutanol and isoamyl alcohol, with ethyl acetate levels aligning with those of the parental strain. The production of lactate ester produced by mutant strains V635mS, V6B12mS, and V6AmS were comparable to that of strain WYm1S, whereas strains V235mS, V2B12mS, and V2AmS exhibited significantly lower lactate ester production than WYm1S. This variation may be attributed to interactions among genes, suboptimal fermentation performance or the knockout of *ILV2*, which may hinder the metabolic effects of exogenous genes *mae1* and *mleS* on L-malate. Further research is needed to elucidate the detailed metabolic mechanisms involved.

### 3.5. Effects of Association Mutation of Genes in S. uvarum on Growth and Fermentation Performance

The growth and fermentation performance of the yeast are key factors influencing the wine quality, in which the growth performance of the strain directly affects the yield of the wine, while fermentation efficiency impacts the wine’s taste, flavor, and overall yield.Thus the physiological characteristic and fermentation performances, such as growth curve, ethanol, residual sugar, and organic acid of wine, were determined to investigate the effects the of the heterologous expression of genes *mae1* and *mleS* in various low-yielding diacetyl *S. uvarums*.

**Figure 8:**
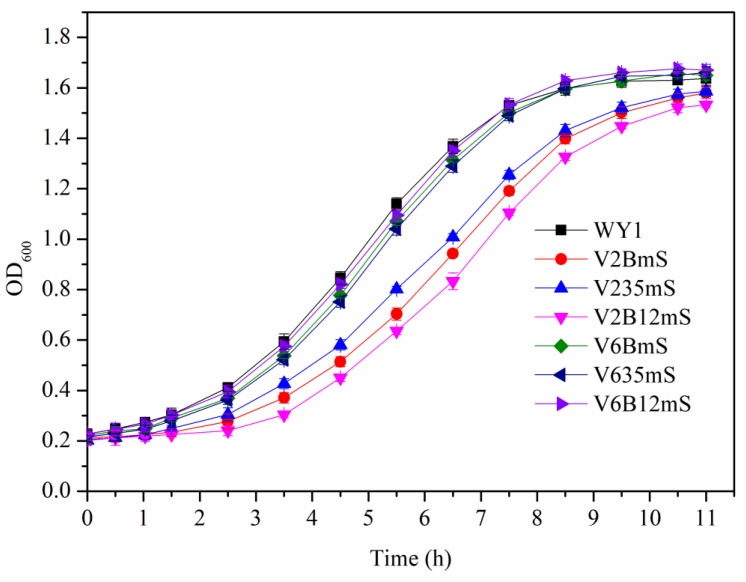
The growth curve of the parental strain and the mutants. (Error bars represent the SD of the average values. Statistical significance *p* < 0.05).

**Figure 9:**
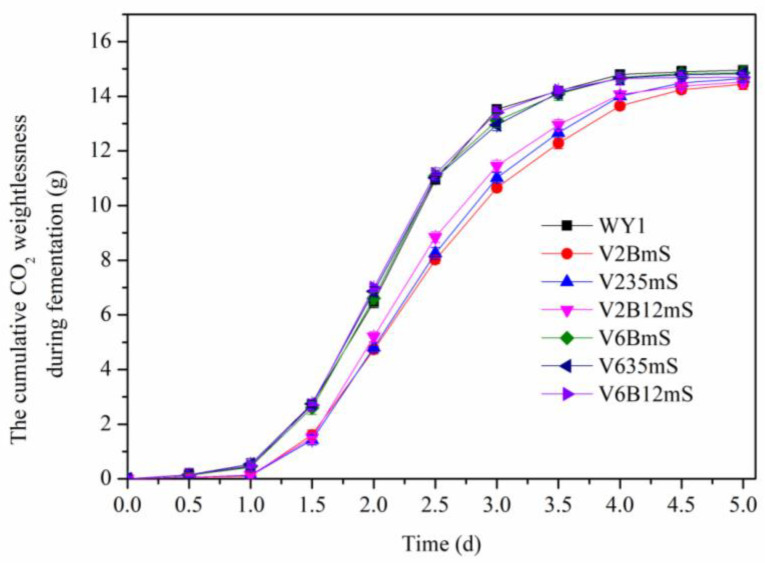
The cumulative CO_2_ weightlessness of the wine fermented by the parental strain and the mutants. (Error bars represent the SD of the average values. Statistical significance *p* < 0.05).

The growth curves were detected after the yeast cells were cultured at 30 °C in YEPD medium, and Wine fermentation rate was conducted for the strains with weight measurements taken every 12 h to calculate CO_2_ release during fermentation. As shown in Figure 8 and Figure 9, the growth and CO_2_ releases trends of mutant strains V6AmS, V635mS, and V6B12mS are similar to those of the parent strain WY1, indicating that the expression of the mae1 gene from *S. pombe* and mleS from *L. lactis* in various low-yielding diacetyl *S. uvarums* did not affect the basic growth and fermentation rate of the mutants. In contrast, mutant strains V2AmS, V235mS, and V2B12mS lag significantly behind the other strains, reaching the growth stabilization phase later and exhibiting lower final cell densities and total CO_2_ release compared to the parental strain.

The ethanol, residual sugar, and organic acid contents in wine were analyzed after fermentation as shown in Table 3. After AF, the residual sugar, ethanol content, tartaric acid, and acetic acid levels produced by mutant strains V6AmS, V635mS, and V6B12mS were similar to those of the parental strain WY1. However, the pH value increased significantly, likely due to the reduced concentration of sharp L-malic acid in the wine, which weakens the wine’s acidity. Additionally, the production of citric acid and succinic acid slightly decreased in V6AmS, V635mS, and V6B12mS. This change may be attributed to the heterologous expression of the *mae1* gene from *S. pombe* in *S. uvarum*, which facilitates the transport of L-malic acid from the extracellular environment into the cell, thus affecting the tricarboxylic acid cycle, (TCA cycle) and altering the levels of citric and succinic acids. In contrast, strains V2AmS, V235mS, and V2B12mS exhibited higher residual sugar and lower alcohol content compared to the parent strain. This phenomenon is likely due to the influence of the exogenous genes *mae1* and *mleS* on the growth rate and metabolic rate of the mutants. It is also possible that excessive genetic modifications adversely affect the growth of the strains, thereby hindering product synthesis.

## 4. Conclusions

Diacetyl is a crucial aroma byproduct with a low threshold level in the AF and MLF processes of wine. However, its concentration can easily exceed the threshold following unmanageable LAB fermentation. This study found that WYmS and WYm1m2 in previous research could effectively degrade malic acid but led to a significant increase in diacetyl levels. Thus, the effect of association mutation of genes *ILV2*, *ILV6*, *ILV3*, *ILV5*, *BDH1*, *BDH2*, *aldB* from *L. plantarum*, *mae1* from *S. pombe* and *mleS* from *L. lactis* in *S. uvarums* on diacetyl, L-malic acid, and other flavors (organic acids, higher alcohols and esters) matebolism was explored, constructing the biological pathway of deacidification and low-yield diacetyl into *S. uvarum*. Of all the mutants, strain V6AmS, V635mS, V6B12mS showed the best effect on deacidification, diacetyl reduction, and flavor balance in wine.

Our work settled several important questions related to mitigating the negative effects of LAB fermentation and improvement of the flavor quality in wine and, leading to further advances in winemaking and broadening applications in scientific investigations. In future studies, we will integrate transcriptomic, proteomic metabolomic analyses to investigate the effects of association mutations and gene interactions on global regulation in *S. uvarum*. Additionally, pilot-scale fermentation will be conducted using 50 L, 200 L, and 500 L fermentors, followed by sensory evaluation of the resulting wine, thereby laying the foundation for the industrial application of these strains.

## Figures and Tables

**Table 1:** Strains and plasmids used in the current study.

Strains or Plasmids	Relevant Characteristic	Reference or Source
Strain		
*E. coli* DH5α	supE44ΔlacU169 (ϕ80lacZΔM15) hsdR17 recAl endAl gyrA96 thi-1 relA	This lab
WY1	Wild-type industrial *Saccharomyces uvarum*	This lab (the parenal strain)
*Lactococcus lactis*	Wild-type industrial *Lactococcus lactis*	This lab
*Lactobacillus plantarum*	Wild-type industrial *Lactobacillus plantarum*	This lab
*Schizosaccharomyces pombe*	Wild-type industrial *Schizosaccharomyces pombe*	This lab
WYS	*PGK1p-mleS* (*Lactococcus lactis*)*-PGK1t-loxP-KanMX-loxP*	Previous study [32]
WYm1	*PGK1p-mae1* (*Schizosaccharomyces pombe*)*-PGK1t-loxP-KanMX-loxP*	Previous study [32]
WYm1S	*PGK1p-mae1* (*Schizosaccharomyces pombe*)*-PGK1t-PGK1p-mleS_N_* (*Lactococcus lactis*)*-PGK1t-loxP-* *KanMX-loxP*	Previous study [32]
V22	*ILV2 (n-2)/*Δ*ilv2*	Previous study [18]
V61	*ILV6 (n-1)/*Δ*ilv6*	Previous study [19]
V6A	*ILV6 (n-2)/*Δ*ilv6*, *PGK1p-aldB-PGK1-loxP-KanMX-loxP*	Previous study [19]
V235	*ILV2 (n-3)/*Δ*ilv2*, *PGK1p-ILV3-PGK1t-PGK1p-ILV5-PGK1t-loxP-* *KanMX-loxP*	Previous study [18]
V2B12	*ILV2 (n-3)/*Δ*ilv2*, *PGK1p-BDH1-PGK1t-PGK1p-BDH2-PGK1t-loxP-* *KanMX-loxP*	This study
V2A	*ILV2 (n-3)/*Δ*ilv2*, *PGK1p-aldB-PGK1-loxP-KanMX-loxP*	This study
V635	*ILV6 (n-2)/*Δ*ilv6*, *PGK1p-ILV3-PGK1t-PGK1p-ILV5-PGK1t-loxP-* *KanMX-loxP*	This study
V6B12	*ILV6 (n-2)/*Δ*ilv6*, *PGK1p-BDH1-PGK1t-PGK1p-BDH2-PGK1t-loxP-* *KanMX-loxP*	This study
V2mSK	*ILV2 (n-3)/*Δ*ilv2*, *PGK1p-mae1-PGK1t-PGK1p-mleS_NZ_-PGK1t-loxP-loxP-* *KanMX-loxP*	This study
V2mS	*ILV2 (n-3)/*Δ*ilv2*, *PGK1p-mae1-PGK1t-PGK1p-mleS_NZ_-PGK1t*	This study
V235mS	*ILV2 (n-3)/*Δ*ilv2*, *PGK1p-mae1-PGK1t-PGK1p-mleS_NZ_-PGK1t-loxP*, *PGK1p-ILV3-PGK1t-PGK1p-ILV5-PGK1t-loxP-KanMX-loxP*	This study
V2AmS	*ILV2 (n-3)/*Δ*ilv2*, *PGK1p-mae1-PGK1t-PGK1p-mleS_NZ_-PGK1t-loxP*, *PGK1p-ALDB-PGK1-loxP-KanMX-loxP*	This study
V2B12mS	*ILV2 (n-3)/*Δ*ilv2*, *PGK1p-mae1-PGK1t-PGK1p-mleS_NZ_-PGK1t-loxP*, *PGK1p-BDH1-PGK1t-PGK1p-BDH2-PGK1t-loxP-KanMX-loxP*	This study
V6mSK	*ILV6 (n-2)/*Δ*ilv6*, *PGK1p-mae1-PGK1t-PGK1p-mleS_NZ_-PGK1t-loxP-KanMX-loxP*	This study
V6mS	*ILV6 (n-2)/*Δ*ilv6*, *PGK1p-mae1-PGK1t-PGK1p-2mleS_NZ_-PGK1t*	This study
V635mS	*ILV6 (n-2)/*Δ*ilv6*, *PGK1p-mae1-PGK1t-PGK1p-mleS_NZ_-PGK1t-loxP*, *PGK1p-ILV3-PGK1t-PGK1p-ILV5-PGK1t-loxP-KanMX-loxP*	This study
V6AmS	*ILV6 (n-2)/*Δ*ilv6*, *PGK1p-mae1-PGK1t-PGK1p-mleS_NZ_-PGK1t-loxP*, *PGK1p-ALDB-PGK1-loxP-KanMX-loxP*	This study
Plasmids		
Yep352 pUG6	Ap^r^, cloning vector *E. coli*/*S. cerevisiae* shuttle vector, containing *Amp*^+^ and *loxP-KanMX-loxP* cassette	This lab This lab
pPGK1	*E. coli*/*S. cerevisiae* shuttle vector, containing *Amp*^+^ and *PGK1p* and *PGK1t*	This lab
pSH-Zeocin	Zeor, Cre expression vector	This lab
Yep-Pm1SK	*Amp*^r^, *Kan*^r^, *PGK1p-mae1-PGK1t-PGK1p-mleS-PGK1t-loxP-KanMX-loxP*	Previous study [32]
Yep-PAK	*Amp*^r^, *Kan*^r^, *PGK1p-aldB-PGK1t-loxP-KanMX-loxP*	Previous study [19]
Yep-PB1B2K	*Amp*^r^, *Kan*^r^, *PGK1p-BDH1-PGK1t-PGK1p-BDH2-PGK1t-loxP-KanMX-loxP*	Previous study [17]
Yep-PV35K	*Amp*^r^, *Kan*^r^, *PGK1p-ILV3-PGK1t-PGK1p-ILV5-PGK1t-loxP-KanMX-loxP*	Previous study [18]

**Table 2:** Primers used in the present study.

Primers	Sequence (5′→3′)
PGK-U	CGCGGATCCTCTAACTGATCTATCCAAAACTG
PGK-D	ACGCGTCGACTAACGAACGCAGAATTTTCGAG
MAE-U	GAATTCCAGATCTCCTCGAGATGCTTAGAACCAGACTATCCG
MAE-D	TCTATCGCAGATCCCTCGAGCTACAATTGGTTGGTGTGCAC
mae2-U	GAATTCCAGATCTCCTCGAGGCACGTGGACCGTCTTACC
mae2-D	TCTATCGCAGATCCCTCGAGAGTTGATGAATAACAATAGGAGAAA
mleS_NZ_-U	GAATTCCAGATCTCCTCGAGATGCGTGCACATGAAATTT
mleS_NZ_-D	TCTATCGCAGATCCCTCGAGTTAGTACTCTGGATACCATTTAAGA
K(ApaI)-U	CCGCTAACAATACCTGGGCCCCAGCTGAAGCTTCGTACGC
K(ApaI)-D	GCACACGGTGTGGTGGGCCCGCATAGGCCACTAGTGGATCTG
mae1-U	GAATTCCAGATCTCCTCGAGTTCATTTTCTCTCTTGGCCAC
mae1-D	TCTATCGCAGATCCCTCGAGCTTTTGTCATGAAATCCCTCTTA
mleP_NZ_-U	GAATTCCAGATCTCCTCGAGATGAAAAAACTTAAAGAAACGA
mleP_NZ_-D	TCTATCGCAGATCCCTCGAGTTAATAAAAGAATCGTATAAGAATT
PGK(SmaI)-U	GTACCCGGGTCTAACTGATCTATCCAAAACTGA
PGK(SmaI)-D	GATCCCCGGGTAACGAACGCAGAATTTTC
GAL80A-U	GTGCCTCTATGATGGGTATG
GAL80A-D	TACCGAGCTCGAATTCGTAATAAGAACGGGAAACCAACTATC
GAL80B-U	TCCACTAGTGGCCTATGCACCTTGATGGATGCTCTGATA
GAL80B-D	ATTCCTGGAGAACCACCTAA
ReGAL80-U	GATAGTTGGTTTCCCGTTCTTATTACGAATTCGAGCTCGGTA
ReGAL80-D	TATCAGAGCATCCATCAAGGTGCATAGGCCACTAGTGGAT
V2m1SA-U	CGTTATTACAGTGCGTCTC
V2m1SA-D	TACCGAGCTCGAATTCGTAATCCCAGAAGTAACCAAGACAA
V2m1SB-U	ATCCACTAGTGGCCTATGCGCCATCGGTGCTCAAGTT
V2m1SB-D	GCTACCACCTGCCACCA
V2m1S-U	TTGTCTTGGTTACTTCTGGGATTACGAATTCGAGCTCGGTA
V2m1S-D	AACTTGAGCACCGATGGCGCATAGGCCACTAGTGGAT
K-U	CAGCTGAAGCTTCGTACGC
K-D	GCATAGGCCACTAGTGGATCTG
ApaI-U	CCTGCTTCAAACCGCTAACAATA
ApaI-D	CGAATGCACACGGTGTGGT
SmaI-U	TTCGAGCTCGGTACCCG
SmaI-D	AGTTAGAGGATCCCCGGG
1YGAL80-U	GATCATCGTAGTGCCCAATT
1YGAL80-D	GTACCGAGCTCGAATTCGT
2YGAL80-U	GGTTTGGTTGATGCGAGTG
2YGAL80-D	CCATTCATCGTGTTGTTTTGG
1YV2m1S-U	TGTAAACAGAACTTTGCCACTA
1YV2m1S-D	GTACCGAGCTCGAATTCGT
2YV2m1S-U	GGTTTGGTTGATGCGAGTG
2YV2m1S-D	TTCATTTATGGCTTCGTCGGA

**Table 3:** Fermentation performances of the mutant strains and the parental strain in the wine fermentation ^a^.

Strain	WY1	V235mS	V2B12mS	V2AmS	V635mS	V6B12mS	V6AmS
Residual sugar (g/L)	2.57 ± 0.10	2.96 ± 0.14 ^a^	2.96 ± 0.08 ^a^	3.11 ± 0.05 ^a^	2.66 ± 0.08	2.61 ± 0.15	2.57 ± 0.25
Ethanol (%, *v*/*v*)	11.03 ± 0.02	10.52 ± 0.05 ^a^	10.45 ± 0.17 ^a^	10.70 ± 0.11 ^a^	11.02 ± 0.15	11.05 ± 0.10	11.00 ± 0.21
pH	2.88 ± 0.11	2.80 ± 0.10	2.83 ± 0.06	2.85 ± 0.15	3.20 ± 0.20 ^a^	3.23 ± 0.12 ^a^	3.25 ± 0.18 ^a^
Weight losses of CO_2_ (g)	14.97 ± 0.10	14.65 ± 0.14^a^	14.52 ± 0.20^a^	14.44 ± 0.20 ^a^	14.92 ± 0.06	14.97 ± 0.10	14.95 ± 0.18
L-malic acid (g/L)	3.661 ± 0.026	3.067 ± 0.010	3.056 ± 0.031	3.069 ± 0.012	1.266 ± 0.008 ^a^	1.183 ± 0.011 ^a^	1.190 ± 0.021 ^a^
L-lactic acid (g/L)	0.144 ± 0.003	0.745 ± 0.003	1.672 ± 0.041	0.737 ± 0.002	3.453 ± 0.024 ^a^	4.405 ± 0.074 ^a^	3.465 ± 0.068 ^a^
Citric acid (g/L)	0.771 ± 0.004	0.770 ± 0.001	0.782 ± 0.002	0.759 ± 0.012	0.750 ± 0.009 ^a^	0.743 ± 0.015 ^a^	0.752 ± 0.002 ^a^
Tartaric Acid (g/L)	2.394 ± 0.055	2.486 ± 0.094	2.414 ± 0.094	2.384 ± 0.087	2.620 ± 0.213	2.376 ± 0.040	2.488 ± 0.019
Succinic acid	1.324 ± 0.014	1.248 ± 0.014	1.233 ± 0.031	1.200 ± 0.018	1.016 ± 0.037 ^a^	0.996 ± 0.014 ^a^	0.967 ± 0.026 ^a^
acetic acid (g/L)	0.193 ± 0.006	0.188 ± 0.008	0.191 ± 0.012	0.176 ± 0.012	0.201 ± 0.006	0.193 ± 0.024	0.207 ± 0.016

^a^ Data are the average of three independent experiments ± the standard deviation. Significant difference of the mutants and the parental strain was confirmed by Student’s *t*-test (*p* < 0.05, n = 3).

## Data Availability

The original contributions presented in the study are included in the article, further inquiries can be directed to the corresponding author.
